# Supplementary material for: Experimental evidence that group size generates divergent benefits of cooperative breeding for male and female ostriches
Source: eLife. 2022 Oct 4;11:e77170. doi: 10.7554/eLife.77170 (PMC9531942; doi:10.7554/eLife.77170)
Supplement: Supplementary file 1. — A: The composition of breeding groups observed in the Karoo National Park. B: Group size effects on the number of eggs produced by males. C: Group size effects on the number of eggs produced by females. D: Group size effects on the number of chicks produced by males. E: Group size effects on the number of chicks produced by females. F: Group size effects on the time nests were incubated. G: The effect of nest incubation on hatching success. H: Group size effects on the amount of time males spent incubating. I: Group size effects on the amount of time females spent incubating. J: Group size effects on the interruptions to incubation. K: The effect of the disparity in incubation on the number of interruptions. L: The effect of the disparity in incubation between males and females on % of eggs broken. M: The effect of the % of eggs broken on hatching success. N: Group size effects on the number of eggs produced by groups. O: Group size effects on the number of chicks produced by groups. P: The effect of average within-group relatedness on the number of chicks produced by males. Q: The effect of average within-group relatedness on the number of chicks produced by females. R: Sample size of experiment and summary statistics of reproductive success and incubation. S: Overview of replacements and removals of individuals in experimental groups. [file elife-77170-supp1.zip › Supplementary file 1.html]

WrestlerSI\_elife\_second.knit


# Supplementary file 1 for “Experimental evidence that group size drives divergent benefits of cooperative breeding for male and female ostriches”

Julian Melgar1, Mads F. Schou1, Maud
Bonato2, Zanell Brand3, Anel
Engelbrecht3, Schalk Cloete4,3 &
Charlie K. Cornwallis1

1 Department of Biology, Lund University, Lund,
Sweden  
2 Kynsna Elephant Park, Kynsna, South Africa  
3 Directorate Animal Sciences, Western Cape Department of
Agriculture, Elsenburg, South Africa  
4 Department of Animal Sciences, University of Stellenbosch,
Matieland, South Africa

\*Corresponding authors:  
Email: julian.melgar@biol.lu.se, charlie.cornwallis@biol.lu.se

This file includes Tables S1-S19

# Supplementary Tables

# **A**: The composition of breedinng groups of adults observed in the karoo national park

|  | Year | Date | Group\_size | Females | Males | male\_female | Sex\_ratio (M:F) |
| --- | --- | --- | --- | --- | --- | --- | --- |
| 1 | 2018 | 17-Nov | 3 | 2 | 1 | 1:2 | 2.0 |
| 2 | 2018 | 17-Nov | 5 | 3 | 2 | 2:3 | 1.5 |
| 5 | 2018 | 17-Nov | 8 | 3 | 5 | 5:3 | 0.6 |
| 7 | 2018 | 18-Nov | 3 | 2 | 1 | 1:2 | 2.0 |
| 8 | 2018 | 18-Nov | 11 | 6 | 5 | 5:6 | 1.2 |
| 9 | 2018 | 18-Nov | 3 | 2 | 1 | 1:2 | 2.0 |
| 10 | 2018 | 18-Nov | 3 | 1 | 2 | 2:1 | 0.5 |
| 17 | 2018 | 18-Nov | 3 | 1 | 2 | 2:1 | 0.5 |
| 19 | 2018 | 18-Nov | 2 | 1 | 1 | 1:1 | 1.0 |
| 20 | 2018 | 18-Nov | 3 | 2 | 1 | 1:2 | 2.0 |
| 21 | 2018 | 18-Nov | 5 | 1 | 4 | 4:1 | 0.2 |
| 22 | 2018 | 18-Nov | 6 | 4 | 2 | 2:4 | 2.0 |
| 24 | 2018 | 18-Nov | 4 | 2 | 2 | 2:2 | 1.0 |
| 25 | 2018 | 18-Nov | 6 | 4 | 2 | 2:4 | 2.0 |
| 26 | 2018 | 18-Nov | 4 | 3 | 1 | 1:3 | 3.0 |
| 27 | 2018 | 18-Nov | 3 | 2 | 1 | 1:2 | 2.0 |
| 29 | 2018 | 18-Nov | 6 | 3 | 3 | 3:3 | 1.0 |
| 30 | 2018 | 18-Nov | 7 | 4 | 3 | 3:4 | 1.3 |
| 34 | 2018 | 19-Nov | 5 | 2 | 3 | 3:2 | 0.7 |
| 39 | 2018 | 19-Nov | 3 | 1 | 2 | 2:1 | 0.5 |
| 43 | 2018 | 19-Nov | 2 | 1 | 1 | 1:1 | 1.0 |
| 45 | 2018 | 19-Nov | 4 | 2 | 2 | 2:2 | 1.0 |
| 47 | 2018 | 19-Nov | 3 | 2 | 1 | 1:2 | 2.0 |
| 49 | 2018 | 19-Nov | 2 | 1 | 1 | 1:1 | 1.0 |
| 50 | 2018 | 19-Nov | 3 | 1 | 2 | 2:1 | 0.5 |
| 51 | 2018 | 19-Nov | 6 | 2 | 4 | 4:2 | 0.5 |
| 52 | 2018 | 19-Nov | 17 | 5 | 12 | 12:5 | 0.4 |
| 53 | 2018 | 19-Nov | 4 | 2 | 3 | 3:2 | 0.7 |
| 54 | 2018 | 19-Nov | 18 | 12 | 6 | 6:12 | 2.0 |
| 55 | 2018 | 19-Nov | 11 | 9 | 2 | 2:9 | 4.5 |
| 57 | 2018 | 19-Nov | 8 | 6 | 2 | 2:6 | 3.0 |
| 58 | 2018 | 19-Nov | 6 | 4 | 2 | 2:4 | 2.0 |
| 60 | 2018 | 19-Nov | 5 | 3 | 2 | 2:3 | 1.5 |
| 61 | 2018 | 19-Nov | 2 | 1 | 1 | 1:1 | 1.0 |
| 62 | 2018 | 19-Nov | 4 | 1 | 3 | 3:1 | 0.3 |
| 64 | 2018 | 19-Nov | 2 | 1 | 1 | 1:1 | 1.0 |
| 65 | 2018 | 19-Nov | 2 | 1 | 1 | 1:1 | 1.0 |
| 66 | 2018 | 19-Nov | 4 | 1 | 3 | 3:1 | 0.3 |
| 67 | 2014 | 08-Nov | 2 | 1 | 1 | 1:1 | 1.0 |
| 68 | 2014 | 08-Nov | 2 | 1 | 1 | 1:1 | 1.0 |
| 69 | 2014 | 08-Nov | 2 | 1 | 1 | 1:1 | 1.0 |
| 72 | 2014 | 08-Nov | 6 | 3 | 3 | 3:3 | 1.0 |
| 73 | 2014 | 08-Nov | 6 | 1 | 5 | 5:1 | 0.2 |
| 74 | 2014 | 08-Nov | 5 | 3 | 2 | 2:3 | 1.5 |
| 76 | 2014 | 08-Nov | 2 | 1 | 1 | 1:1 | 1.0 |
| 78 | 2014 | 08-Nov | 5 | 1 | 4 | 4:1 | 0.2 |
| 79 | 2014 | 08-Nov | 4 | 2 | 2 | 2:2 | 1.0 |
| 80 | 2014 | 08-Nov | 4 | 1 | 3 | 3:1 | 0.3 |
| 81 | 2014 | 08-Nov | 6 | 3 | 3 | 3:3 | 1.0 |
| 82 | 2014 | 08-Nov | 3 | 2 | 1 | 1:2 | 2.0 |
| 84 | 2014 | 08-Nov | 15 | 12 | 3 | 3:12 | 4.0 |
| 85 | 2014 | 08-Nov | 12 | 9 | 3 | 3:9 | 3.0 |
| 86 | 2014 | 08-Nov | 2 | 1 | 1 | 1:1 | 1.0 |
| 87 | 2014 | 08-Nov | 8 | 4 | 4 | 4:4 | 1.0 |
| 88 | 2014 | 08-Nov | 2 | 1 | 1 | 1:1 | 1.0 |
| 89 | 2014 | 08-Nov | 4 | 3 | 1 | 1:3 | 3.0 |
| 90 | 2014 | 08-Nov | 3 | 1 | 2 | 2:1 | 0.5 |
| 94 | 2014 | 08-Nov | 2 | 1 | 1 | 1:1 | 1.0 |
| 95 | 2014 | 09-Nov | 2 | 1 | 1 | 1:1 | 1.0 |
| 96 | 2014 | 09-Nov | 6 | 4 | 2 | 2:4 | 2.0 |
| 101 | 2014 | 09-Nov | 4 | 2 | 2 | 2:2 | 1.0 |
| 103 | 2014 | 09-Nov | 6 | 4 | 2 | 2:4 | 2.0 |
| 106 | 2014 | 09-Nov | 2 | 1 | 1 | 1:1 | 1.0 |
| 107 | 2014 | 09-Nov | 2 | 1 | 1 | 1:1 | 1.0 |
| 108 | 2014 | 09-Nov | 7 | 1 | 6 | 6:1 | 0.2 |

# **B**: Group size effects on the number of eggs produced by males

| Fixed Effects | Posterior Mode (CI) | pMCMC |
| --- | --- | --- |
| No\_care : Nmales\_3 | 0.46 (0.13, 0.65) |  |
| No\_care : Nmales\_1 | 1.65 (1.32, 1.84) |  |
| Care : Nmales\_3 | 0.11 (-0.17, 0.38) |  |
| Care : Nmales\_1 | 1.05 (0.81, 1.39) |  |
| No\_care : Nfemales | 0.55 (0.38, 0.73) | 0.001 |
| Care : Nfemales | 0.38 (0.24, 0.54) | 0.001 |
| No\_care : Nfemales^2 | -0.21 (-0.37, -0.03) | 0.012 |
| Care : Nfemales^2 | 0.11 (-0.06, 0.26) | 0.252 |
| No\_care : Nfemales : Nmales\_3 | 0.5 (0.28, 0.83) | 0.001 |
| No\_care : Nfemales : Nmales\_1 | 0.56 (0.32, 0.79) | 0.001 |
| Care : Nfemales : Nmales\_3 | 0.34 (0.13, 0.55) | 0.002 |
| Care : Nfemales : Nmales\_1 | 0.38 (0.22, 0.66) | 0.001 |
| No\_care : Nfemales^2 : Nmales\_3 | -0.19 (-0.46, 0.05) | 0.14 |
| No\_care : Nfemales^2 : Nmales\_1 | -0.25 (-0.45, -0.01) | 0.03 |
| Care : Nfemales^2 : Nmales\_3 | 0 (-0.18, 0.28) | 0.654 |
| Care : Nfemales^2 : Nmales\_1 | 0.16 (-0.1, 0.37) | 0.29 |
| Fixed Effect Comparisons | Posterior Mode (CI) | pMCMC |
| No\_care : Nmales\_3 vs No\_care : Nmales\_1 | -1.14 (-1.49, -0.96) | 0.001 |
| Care : Nmales\_3 vs Care : Nmales\_1 | -1 (-1.24, -0.67) | 0.001 |
| No\_care : Nfemales vs Care : Nfemales | 0.14 (-0.04, 0.41) | 0.146 |
| No\_care : Nfemales^2 vs Care : Nfemales^2 | -0.28 (-0.5, -0.07) | 0.004 |
| No\_care : Nfemales : Nmales\_3 vs No\_care : Nfemales : Nmales\_1 | -0.03 (-0.38, 0.33) | 0.918 |
| No\_care : Nfemales : Nmales\_3 vs Care : Nfemales : Nmales\_3 | 0.14 (-0.14, 0.54) | 0.248 |
| No\_care : Nfemales : Nmales\_1 vs Care : Nfemales : Nmales\_1 | 0.17 (-0.18, 0.44) | 0.414 |
| Care : Nfemales : Nmales\_3 vs Care : Nfemales : Nmales\_1 | -0.02 (-0.38, 0.22) | 0.566 |
| No\_care : Nfemales^2 : Nmales\_3 vs No\_care : Nfemales^2 : Nmales\_1 | 0.03 (-0.27, 0.38) | 0.736 |
| No\_care : Nfemales^2 : Nmales\_3 vs Care : Nfemales^2 : Nmales\_3 | -0.23 (-0.56, 0.12) | 0.172 |
| No\_care : Nfemales^2 : Nmales\_1 vs Care : Nfemales^2 : Nmales\_1 | -0.25 (-0.68, -0.07) | 0.018 |
| Care : Nfemales^2 : Nmales\_3 vs Care : Nfemales^2 : Nmales\_1 | -0.12 (-0.41, 0.22) | 0.66 |
| Random Effects | Posterior Mode (CI) | I2 % (CI) |
| Year | 0 (0, 0.05) | 41.69 (2.34, 90.34) |
| Camp | 0 (0, 0.02) | 33.85 (0.36, 79.74) |
| Residual | 0 (0, 0.01) | 24.45 (0.58, 68.73) |

# **C**: Group size effects on the number of eggs produced by females

| Fixed Effects | Posterior Mode (CI) | pMCMC |
| --- | --- | --- |
| No\_care : Nmales\_3 | 1.56 (1.41, 1.73) |  |
| No\_care : Nmales\_1 | 1.62 (1.4, 1.83) |  |
| Care : Nmales\_3 | 1.3 (1.13, 1.48) |  |
| Care : Nmales\_1 | 1.21 (0.94, 1.4) |  |
| No\_care : Nfemales | -0.08 (-0.18, 0) | 0.03 |
| Care : Nfemales | -0.2 (-0.28, -0.11) | 0.001 |
| No\_care : Nfemales^2 | 0.01 (-0.1, 0.09) | 0.898 |
| Care : Nfemales^2 | 0.22 (0.12, 0.32) | 0.001 |
| No\_care : Nfemales : Nmales\_3 | -0.12 (-0.22, 0.01) | 0.088 |
| No\_care : Nfemales : Nmales\_1 | -0.08 (-0.25, 0.07) | 0.308 |
| Care : Nfemales : Nmales\_3 | -0.2 (-0.31, -0.1) | 0.002 |
| Care : Nfemales : Nmales\_1 | -0.19 (-0.34, -0.05) | 0.012 |
| No\_care : Nfemales^2 : Nmales\_3 | 0.01 (-0.09, 0.13) | 0.798 |
| No\_care : Nfemales^2 : Nmales\_1 | -0.12 (-0.22, 0.11) | 0.492 |
| Care : Nfemales^2 : Nmales\_3 | 0.2 (0.08, 0.3) | 0.001 |
| Care : Nfemales^2 : Nmales\_1 | 0.26 (0.1, 0.49) | 0.002 |
| Fixed Effect Comparisons | Posterior Mode (CI) | pMCMC |
| No\_care : Nmales\_3 vs No\_care : Nmales\_1 | -0.07 (-0.25, 0.16) | 0.686 |
| Care : Nmales\_3 vs Care : Nmales\_1 | 0.13 (-0.11, 0.31) | 0.306 |
| No\_care : Nfemales vs Care : Nfemales | 0.1 (-0.03, 0.21) | 0.112 |
| No\_care : Nfemales^2 vs Care : Nfemales^2 | -0.22 (-0.36, -0.1) | 0.001 |
| No\_care : Nfemales : Nmales\_3 vs No\_care : Nfemales : Nmales\_1 | -0.04 (-0.21, 0.17) | 0.822 |
| No\_care : Nfemales : Nmales\_3 vs Care : Nfemales : Nmales\_3 | 0.13 (-0.03, 0.25) | 0.172 |
| No\_care : Nfemales : Nmales\_1 vs Care : Nfemales : Nmales\_1 | 0.11 (-0.1, 0.3) | 0.318 |
| Care : Nfemales : Nmales\_3 vs Care : Nfemales : Nmales\_1 | 0.02 (-0.19, 0.17) | 0.844 |
| No\_care : Nfemales^2 : Nmales\_3 vs No\_care : Nfemales^2 : Nmales\_1 | 0.02 (-0.13, 0.28) | 0.45 |
| No\_care : Nfemales^2 : Nmales\_3 vs Care : Nfemales^2 : Nmales\_3 | -0.18 (-0.34, -0.02) | 0.03 |
| No\_care : Nfemales^2 : Nmales\_1 vs Care : Nfemales^2 : Nmales\_1 | -0.34 (-0.61, -0.11) | 0.006 |
| Care : Nfemales^2 : Nmales\_3 vs Care : Nfemales^2 : Nmales\_1 | -0.13 (-0.33, 0.13) | 0.328 |
| Random Effects | Posterior Mode (CI) | I2 % (CI) |
| Year | 0 (0, 0.02) | 33.91 (1.59, 79.02) |
| Camp | 0 (0, 0.02) | 40.26 (2.27, 81.64) |
| Residual | 0 (0, 0.01) | 25.83 (1.07, 65.2) |

# **D**: Group size effects on the number of chicks produced by males

| Fixed Effects | Posterior Mode (CI) | pMCMC |
| --- | --- | --- |
| No\_care : Nmales\_3 | -0.12 (-0.58, 0.22) |  |
| No\_care : Nmales\_1 | 1.08 (0.69, 1.52) |  |
| Care : Nmales\_3 | -0.95 (-1.45, -0.43) |  |
| Care : Nmales\_1 | -0.18 (-0.79, 0.33) |  |
| No\_care : Nfemales | 0.54 (0.26, 0.79) | 0.001 |
| Care : Nfemales | 0.64 (0.34, 1.14) | 0.001 |
| No\_care : Nfemales^2 | -0.32 (-0.51, -0.04) | 0.026 |
| Care : Nfemales^2 | -0.14 (-0.49, 0.21) | 0.462 |
| No\_care : Nfemales : Nmales\_3 | 0.68 (0.22, 1.02) | 0.001 |
| No\_care : Nfemales : Nmales\_1 | 0.47 (0.17, 0.86) | 0.004 |
| Care : Nfemales : Nmales\_3 | 0.52 (0.11, 0.9) | 0.006 |
| Care : Nfemales : Nmales\_1 | 2.23 (0.72, 4.49) | 0.001 |
| No\_care : Nfemales^2 : Nmales\_3 | -0.34 (-0.63, 0.09) | 0.148 |
| No\_care : Nfemales^2 : Nmales\_1 | -0.41 (-0.6, 0.01) | 0.022 |
| Care : Nfemales^2 : Nmales\_3 | 0.19 (-0.32, 0.52) | 0.542 |
| Care : Nfemales^2 : Nmales\_1 | -1.58 (-2.91, -0.33) | 0.004 |
| Fixed Effect Comparisons | Posterior Mode (CI) | pMCMC |
| No\_care : Nmales\_3 vs No\_care : Nmales\_1 | -1.24 (-1.7, -0.86) | 0.001 |
| Care : Nmales\_3 vs Care : Nmales\_1 | -0.53 (-1.13, -0.06) | 0.016 |
| No\_care : Nfemales vs Care : Nfemales | -0.19 (-0.7, 0.18) | 0.352 |
| No\_care : Nfemales^2 vs Care : Nfemales^2 | -0.18 (-0.56, 0.24) | 0.448 |
| No\_care : Nfemales : Nmales\_3 vs No\_care : Nfemales : Nmales\_1 | 0.16 (-0.36, 0.65) | 0.608 |
| No\_care : Nfemales : Nmales\_3 vs Care : Nfemales : Nmales\_3 | 0.04 (-0.39, 0.63) | 0.686 |
| No\_care : Nfemales : Nmales\_1 vs Care : Nfemales : Nmales\_1 | -1.74 (-4.1, -0.22) | 0.008 |
| Care : Nfemales : Nmales\_3 vs Care : Nfemales : Nmales\_1 | -2.12 (-4.05, -0.12) | 0.008 |
| No\_care : Nfemales^2 : Nmales\_3 vs No\_care : Nfemales^2 : Nmales\_1 | 0.2 (-0.4, 0.51) | 0.802 |
| No\_care : Nfemales^2 : Nmales\_3 vs Care : Nfemales^2 : Nmales\_3 | -0.24 (-0.95, 0.1) | 0.122 |
| No\_care : Nfemales^2 : Nmales\_1 vs Care : Nfemales^2 : Nmales\_1 | 0.93 (-0.15, 2.46) | 0.058 |
| Care : Nfemales^2 : Nmales\_3 vs Care : Nfemales^2 : Nmales\_1 | 1.4 (0.24, 2.95) | 0.008 |
| Random Effects | Posterior Mode (CI) | I2 % (CI) |
| Year | 0 (0, 0.08) | 9.35 (0.07, 32.26) |
| Camp | 0.13 (0, 0.34) | 60.03 (13.92, 99.13) |
| Residual | 0 (0, 0.26) | 30.62 (0.1, 79.01) |

# **E**: Group size effects on the number of chicks produced by females

| Fixed Effects | Posterior Mode (CI) | pMCMC |
| --- | --- | --- |
| No\_care : Nmales\_3 | 1.06 (0.74, 1.39) |  |
| No\_care : Nmales\_1 | 1.05 (0.69, 1.5) |  |
| Care : Nmales\_3 | 0.26 (-0.17, 0.61) |  |
| Care : Nmales\_1 | -0.47 (-0.94, 0.08) |  |
| No\_care : Nfemales | -0.1 (-0.28, 0.04) | 0.13 |
| Care : Nfemales | 0.02 (-0.18, 0.21) | 0.942 |
| No\_care : Nfemales^2 | -0.01 (-0.13, 0.19) | 0.84 |
| Care : Nfemales^2 | 0.16 (-0.01, 0.41) | 0.06 |
| No\_care : Nfemales : Nmales\_3 | -0.07 (-0.28, 0.08) | 0.3 |
| No\_care : Nfemales : Nmales\_1 | -0.13 (-0.45, 0.1) | 0.21 |
| Care : Nfemales : Nmales\_3 | -0.12 (-0.29, 0.11) | 0.394 |
| Care : Nfemales : Nmales\_1 | 0.55 (0.04, 1.74) | 0.008 |
| No\_care : Nfemales^2 : Nmales\_3 | 0.06 (-0.09, 0.28) | 0.4 |
| No\_care : Nfemales^2 : Nmales\_1 | -0.16 (-0.41, 0.14) | 0.456 |
| Care : Nfemales^2 : Nmales\_3 | 0.34 (0.08, 0.53) | 0.014 |
| Care : Nfemales^2 : Nmales\_1 | -0.57 (-1.34, 0.03) | 0.04 |
| Fixed Effect Comparisons | Posterior Mode (CI) | pMCMC |
| No\_care : Nmales\_3 vs No\_care : Nmales\_1 | -0.1 (-0.39, 0.35) | 0.856 |
| Care : Nmales\_3 vs Care : Nmales\_1 | 0.65 (0.18, 1.11) | 0.008 |
| No\_care : Nfemales vs Care : Nfemales | -0.13 (-0.37, 0.09) | 0.246 |
| No\_care : Nfemales^2 vs Care : Nfemales^2 | -0.09 (-0.41, 0.08) | 0.178 |
| No\_care : Nfemales : Nmales\_3 vs No\_care : Nfemales : Nmales\_1 | 0.05 (-0.25, 0.37) | 0.712 |
| No\_care : Nfemales : Nmales\_3 vs Care : Nfemales : Nmales\_3 | 0.01 (-0.25, 0.26) | 0.966 |
| No\_care : Nfemales : Nmales\_1 vs Care : Nfemales : Nmales\_1 | -1.08 (-2.03, -0.25) | 0.001 |
| Care : Nfemales : Nmales\_3 vs Care : Nfemales : Nmales\_1 | -0.81 (-1.9, -0.13) | 0.006 |
| No\_care : Nfemales^2 : Nmales\_3 vs No\_care : Nfemales^2 : Nmales\_1 | 0.27 (-0.14, 0.49) | 0.258 |
| No\_care : Nfemales^2 : Nmales\_3 vs Care : Nfemales^2 : Nmales\_3 | -0.23 (-0.5, 0.05) | 0.118 |
| No\_care : Nfemales^2 : Nmales\_1 vs Care : Nfemales^2 : Nmales\_1 | 0.55 (-0.19, 1.28) | 0.15 |
| Care : Nfemales^2 : Nmales\_3 vs Care : Nfemales^2 : Nmales\_1 | 1.01 (0.2, 1.68) | 0.004 |
| Random Effects | Posterior Mode (CI) | I2 % (CI) |
| Year | 0 (0, 0.06) | 3.75 (0.05, 15.39) |
| Camp | 0.12 (0.02, 0.31) | 35.93 (7.11, 63.09) |
| Residual | 0.2 (0.07, 0.39) | 60.32 (30.63, 87.5) |

# **F**: Group size effects on the time nests were incubated

| Fixed Effects | Posterior Mode (CI) | pMCMC |
| --- | --- | --- |
| Nmales\_3 | 0.35 (-0.51, 1.03) |  |
| Nmales\_1 | -1.24 (-1.91, 0.13) |  |
| Number nests | 0.23 (-0.15, 0.62) | 0.19 |
| Nfemales | 0.94 (0.56, 1.34) | 0.001 |
| Nfemales^2 | -0.26 (-0.8, 0.06) | 0.092 |
| Nmales\_3 : Nfemales | 0.78 (0.32, 1.11) | 0.001 |
| Nmales\_1 : Nfemales | 1.56 (1.02, 2.28) | 0.001 |
| Nmales\_3 : Nfemales^2 | -0.09 (-0.54, 0.36) | 0.64 |
| Nmales\_1 : Nfemales^2 | -0.78 (-1.64, -0.26) | 0.014 |
| Fixed Effect Comparisons | Posterior Mode (CI) | pMCMC |
| Nmales\_3 vs Nmales\_1 | 1.27 (0.29, 2.06) | 0.006 |
| Nmales\_3 : Nfemales vs Nmales\_1 : Nfemales | -0.94 (-1.67, -0.14) | 0.024 |
| Nmales\_3 : Nfemales^2 vs Nmales\_1 : Nfemales^2 | 0.62 (-0.01, 1.59) | 0.04 |
| Random Effects | Posterior Mode (CI) | I2 % (CI) |
| Year | 0.01 (0, 1.71) | 5.99 (0.01, 21.46) |
| Camp | 0.01 (0, 0.64) | 2.2 (0, 9.06) |
| Residual | 2.82 (1.98, 3.89) | 42.69 (29.12, 54.01) |

# **G**: The effect of nest incubation on hatching success

| Fixed Effects | Posterior Mode (CI) | pMCMC |
| --- | --- | --- |
| Nmales\_3 | -2.31 (-3.12, -1.53) |  |
| Nmales\_1 | -2.75 (-3.66, -1.44) |  |
| Nest incubation (% time) | 0.35 (0.03, 0.7) | 0.034 |
| Time monitored (days Z) | 0.02 (-0.53, 0.61) | 0.994 |
| Nnests | 0.12 (-0.3, 0.46) | 0.696 |
| Nfemales | 0.01 (-0.36, 0.58) | 0.622 |
| Nfemales^2 | -0.07 (-0.45, 0.52) | 0.956 |
| Nmales\_3 : Nfemales | -0.1 (-0.45, 0.55) | 0.88 |
| Nmales\_1 : Nfemales | 1.21 (-0.11, 2.27) | 0.064 |
| Nmales\_3 : Nfemales^2 | 0.27 (-0.23, 0.76) | 0.27 |
| Nmales\_1 : Nfemales^2 | -1.07 (-2.13, -0.03) | 0.052 |
| Random Effects | Posterior Mode (CI) | I2 % (CI) |
| Year | 0.01 (0, 1.08) | 3.74 (0, 16.53) |
| Camp | 0 (0, 1.21) | 5.44 (0.01, 18.05) |
| Residual | 1.91 (1, 3.36) | 34.41 (20, 50.56) |

# **H**: Group size effects on the amount of time males spent incubating

| Fixed Effects | Posterior Mode (CI) | pMCMC |
| --- | --- | --- |
| Nmales\_3 | -7.67 (-8.83, -6.03) |  |
| Nmales\_1 | -4.6 (-6.77, -2.1) |  |
| Nfemales | 0.09 (-0.62, 1.05) | 0.57 |
| Nfemales^2 | -0.34 (-1.09, 0.46) | 0.502 |
| Nmales\_3 : Nfemales | -0.22 (-0.96, 0.9) | 0.918 |
| Nmales\_1 : Nfemales | 1.97 (-0.35, 3.81) | 0.102 |
| Nmales\_3 : Nfemales^2 | -0.37 (-1.06, 0.63) | 0.618 |
| Nmales\_1 : Nfemales^2 | -1.28 (-3.16, 1.05) | 0.33 |
| Fixed Effect Comparisons | Posterior Mode (CI) | pMCMC |
| Nmales\_3 vs Nmales\_1 | -3.07 (-5.28, -0.76) | 0.004 |
| Nmales\_3 : Nfemales vs Nmales\_1 : Nfemales | -2.04 (-3.92, 0.57) | 0.126 |
| Nmales\_3 : Nfemales^2 vs Nmales\_1 : Nfemales^2 | 1.26 (-1.5, 2.95) | 0.5 |
| Random Effects | Posterior Mode (CI) | I2 % (CI) |
| Year | 0 (0, 1.04) | 0.58 (0, 3.04) |
| Camp | 0 (0, 0.83) | 0.5 (0, 2.41) |
| Group | 0 (0, 0.83) | 0.5 (0, 2.41) |
| ID | 0.02 (0, 6.07) | 3.72 (0, 16.93) |
| Residual | 27.56 (19.9, 39.7) | 85.15 (71.44, 93.11) |

# **I**: Group size effects on the amount of time females spent incubating

| Fixed Effects | Posterior Mode (CI) | pMCMC |
| --- | --- | --- |
| Nmales\_3 | -4.06 (-4.96, -3.06) |  |
| Nmales\_1 | -4.64 (-6.1, -3.66) |  |
| Nfemales | -0.3 (-0.77, 0.18) | 0.212 |
| Nfemales^2 | -0.14 (-0.68, 0.44) | 0.776 |
| Nmales\_3 : Nfemales | -0.46 (-1.03, 0.01) | 0.07 |
| Nmales\_1 : Nfemales | 0.15 (-0.69, 1.05) | 0.634 |
| Nmales\_3 : Nfemales^2 | 0.24 (-0.48, 0.7) | 0.732 |
| Nmales\_1 : Nfemales^2 | -0.38 (-1.53, 0.34) | 0.22 |
| Fixed Effect Comparisons | Posterior Mode (CI) | pMCMC |
| Nmales\_3 vs Nmales\_1 | 1.02 (-0.04, 1.85) | 0.04 |
| Nmales\_3 : Nfemales vs Nmales\_1 : Nfemales | -0.8 (-1.66, 0.17) | 0.134 |
| Nmales\_3 : Nfemales^2 vs Nmales\_1 : Nfemales^2 | 0.61 (-0.33, 1.67) | 0.174 |
| Random Effects | Posterior Mode (CI) | I2 % (CI) |
| Year | 0.06 (0, 1.77) | 2.73 (0, 10.55) |
| Camp | 0.01 (0, 1.88) | 3.45 (0, 11.1) |
| Group | 0.01 (0, 1.45) | 2.08 (0, 8.81) |
| ID | 2.86 (0.86, 5.26) | 17.31 (5.33, 30.28) |
| Residual | 8.51 (6.28, 11.07) | 53.74 (39.69, 67.38) |

# **J**: Group size effects on the interruptions to incubation

| Fixed Effects | Posterior Mode (CI) | pMCMC |
| --- | --- | --- |
| Nmales\_3 | 1.59 (0.78, 2.4) |  |
| Nmales\_1 | -0.14 (-1, 1.25) |  |
| Nfemales | 0.52 (-0.06, 0.95) | 0.06 |
| Nfemales^2 | -0.67 (-1.13, -0.16) | 0.018 |
| Nmales\_3 : Nfemales | 0.44 (-0.11, 0.88) | 0.15 |
| Nmales\_1 : Nfemales | 1.71 (-0.03, 5.47) | 0.034 |
| Nmales\_3 : Nfemales^2 | -0.55 (-1.1, 0) | 0.03 |
| Nmales\_1 : Nfemales^2 | -1.3 (-4.47, 0.17) | 0.048 |
| Fixed Effect Comparisons | Posterior Mode (CI) | pMCMC |
| Nmales\_3 vs Nmales\_1 | 1.5 (0.5, 2.73) | 0.002 |
| Nmales\_3 : Nfemales vs Nmales\_1 : Nfemales | -1.64 (-5.13, 0.5) | 0.106 |
| Nmales\_3 : Nfemales^2 vs Nmales\_1 : Nfemales^2 | 1.23 (-0.55, 4.09) | 0.224 |
| Random Effects | Posterior Mode (CI) | I2 % (CI) |
| Year | 0.01 (0, 0.78) | 4.73 (0.01, 19.29) |
| Camp | 0.01 (0, 1.01) | 5.81 (0.01, 25.69) |
| Residual | 2.89 (1.72, 4.97) | 89.47 (63.25, 99.95) |

# **K**: The effect of the disparity in incubation on the number of interruptions

| Fixed Effects | Posterior Mode (CI) | pMCMC |
| --- | --- | --- |
| Nmales\_3 : Difference in incubation | -1.43 (-2.36, -0.26) | 0.024 |
| Nmales\_1 : Difference in incubation | 0.18 (-1.4, 1.33) | 0.994 |
| Random Effects | Posterior Mode (CI) | I2 % (CI) |
| Year | 0.01 (0, 1.8) | 9.75 (0.01, 32.67) |
| Camp | 0.01 (0, 3.03) | 25.1 (0.01, 52.24) |
| Residual | 2.13 (1.29, 4.88) | 65.15 (32.8, 99.84) |

# **L**: The effect of the disparity in incubation between males and females on % of eggs broken

| Fixed Effects | Posterior Mode (CI) | pMCMC |
| --- | --- | --- |
| Nmales\_3 : Difference in incubation | -0.64 (-1.23, -0.06) | 0.026 |
| Nmales\_1 : Difference in incubation | 0 (-0.73, 0.67) | 0.936 |
| Random Effects | Posterior Mode (CI) | I2 % (CI) |
| Year | 0 (0, 0.57) | 3.94 (0.01, 11.73) |
| Camp | 0.01 (0, 0.39) | 2.6 (0.01, 8.48) |
| Residual | 0.87 (0.51, 1.39) | 20.19 (12.04, 28.72) |

# **M**: The effect of the % of eggs broken on hatching success

| Fixed Effects | Posterior Mode (CI) | pMCMC |
| --- | --- | --- |
| Intercept | -2.49 (-2.86, -2.02) |  |
| Broken eggs (%) | -0.82 (-1.2, -0.58) | 0.001 |
| Random Effects | Posterior Mode (CI) | I2 % (CI) |
| Year | 0 (0, 0.26) | 1.21 (0.01, 5.46) |
| Camp | 0.01 (0, 0.49) | 2.9 (0, 10.15) |
| Residual | 0.85 (0.53, 1.76) | 24.05 (12.96, 34.28) |

# **N**: Group size effects on the number of eggs produced by groups

| Fixed Effects | Posterior Mode (CI) | pMCMC |
| --- | --- | --- |
| No\_care : Nmales\_3 | 4.45 (3.76, 4.85) |  |
| No\_care : Nmales\_1 | 4.79 (4.13, 5.51) |  |
| Care : Nmales\_3 | 3.23 (2.62, 3.74) |  |
| Care : Nmales\_1 | 2.93 (2.05, 3.39) |  |
| No\_care : Nfemales | 1.89 (1.53, 2.13) | 0.001 |
| Care : Nfemales | 1.51 (1.18, 1.78) | 0.001 |
| No\_care : Nfemales^2 | -0.19 (-0.45, 0.13) | 0.304 |
| Care : Nfemales^2 | 0.35 (0.13, 0.7) | 0.01 |
| No\_care : Nfemales : Nmales\_3 | 1.77 (1.51, 2.15) | 0.001 |
| No\_care : Nfemales : Nmales\_1 | 1.99 (1.46, 2.47) | 0.001 |
| Care : Nfemales : Nmales\_3 | 1.53 (1.17, 1.86) | 0.001 |
| Care : Nfemales : Nmales\_1 | 1.53 (0.99, 1.97) | 0.001 |
| No\_care : Nfemales^2 : Nmales\_3 | -0.07 (-0.44, 0.26) | 0.612 |
| No\_care : Nfemales^2 : Nmales\_1 | -0.37 (-0.94, 0.18) | 0.198 |
| Care : Nfemales^2 : Nmales\_3 | 0.3 (-0.03, 0.65) | 0.064 |
| Care : Nfemales^2 : Nmales\_1 | 0.5 (0.07, 1.17) | 0.034 |
| Fixed Effect Comparisons | Posterior Mode (CI) | pMCMC |
| No\_care : Nmales\_3 vs No\_care : Nmales\_1 | -0.41 (-1.16, 0.15) | 0.138 |
| Care : Nmales\_3 vs Care : Nmales\_1 | 0.48 (-0.2, 1.05) | 0.186 |
| No\_care : Nfemales vs Care : Nfemales | 0.38 (-0.04, 0.72) | 0.09 |
| No\_care : Nfemales^2 vs Care : Nfemales^2 | -0.55 (-0.98, -0.18) | 0.008 |
| No\_care : Nfemales : Nmales\_3 vs No\_care : Nfemales : Nmales\_1 | -0.04 (-0.74, 0.47) | 0.586 |
| No\_care : Nfemales : Nmales\_3 vs Care : Nfemales : Nmales\_3 | 0.35 (-0.13, 0.76) | 0.188 |
| No\_care : Nfemales : Nmales\_1 vs Care : Nfemales : Nmales\_1 | 0.56 (-0.15, 1.09) | 0.126 |
| Care : Nfemales : Nmales\_3 vs Care : Nfemales : Nmales\_1 | -0.11 (-0.55, 0.65) | 0.914 |
| No\_care : Nfemales^2 : Nmales\_3 vs No\_care : Nfemales^2 : Nmales\_1 | 0.53 (-0.35, 0.91) | 0.42 |
| No\_care : Nfemales^2 : Nmales\_3 vs Care : Nfemales^2 : Nmales\_3 | -0.31 (-0.86, 0.08) | 0.094 |
| No\_care : Nfemales^2 : Nmales\_1 vs Care : Nfemales^2 : Nmales\_1 | -0.85 (-1.72, -0.24) | 0.012 |
| Care : Nfemales^2 : Nmales\_3 vs Care : Nfemales^2 : Nmales\_1 | -0.27 (-0.87, 0.44) | 0.376 |
| Random Effects | Posterior Mode (CI) | I2 % (CI) |
| Year | 0 (0, 0.19) | 2.08 (0.01, 8.24) |
| Camp | 0.14 (0, 0.55) | 10.27 (0.02, 22.94) |
| Residual | 2 (1.54, 2.39) | 87.65 (73.69, 99.92) |

# **O**: Group size effects on the number of chicks produced by groups

| Fixed Effects | Posterior Mode (CI) | pMCMC |
| --- | --- | --- |
| No\_care : Nmales\_3 | 2.79 (2.19, 3.57) |  |
| No\_care : Nmales\_1 | 3.15 (2.27, 3.93) |  |
| Care : Nmales\_3 | 1.32 (0.72, 2.03) |  |
| Care : Nmales\_1 | 0.74 (-0.11, 1.57) |  |
| No\_care : Nfemales | 1.07 (0.73, 1.47) | 0.001 |
| Care : Nfemales | 0.8 (0.41, 1.13) | 0.001 |
| No\_care : Nfemales^2 | -0.24 (-0.59, 0.13) | 0.272 |
| Care : Nfemales^2 | 0.2 (-0.17, 0.58) | 0.286 |
| No\_care : Nfemales : Nmales\_3 | 1.15 (0.71, 1.57) | 0.001 |
| No\_care : Nfemales : Nmales\_1 | 0.95 (0.41, 1.58) | 0.001 |
| Care : Nfemales : Nmales\_3 | 0.73 (0.4, 1.28) | 0.001 |
| Care : Nfemales : Nmales\_1 | 0.74 (0.08, 1.28) | 0.034 |
| No\_care : Nfemales^2 : Nmales\_3 | 0.08 (-0.49, 0.35) | 0.918 |
| No\_care : Nfemales^2 : Nmales\_1 | -0.81 (-1.31, -0.02) | 0.04 |
| Care : Nfemales^2 : Nmales\_3 | 0.35 (-0.16, 0.76) | 0.158 |
| Care : Nfemales^2 : Nmales\_1 | -0.05 (-0.75, 0.57) | 0.816 |
| Fixed Effect Comparisons | Posterior Mode (CI) | pMCMC |
| No\_care : Nmales\_3 vs No\_care : Nmales\_1 | -0.46 (-1.04, 0.62) | 0.558 |
| Care : Nmales\_3 vs Care : Nmales\_1 | 0.6 (-0.13, 1.47) | 0.124 |
| No\_care : Nfemales vs Care : Nfemales | 0.44 (-0.1, 0.81) | 0.15 |
| No\_care : Nfemales^2 vs Care : Nfemales^2 | -0.27 (-0.94, 0.04) | 0.092 |
| No\_care : Nfemales : Nmales\_3 vs No\_care : Nfemales : Nmales\_1 | 0.27 (-0.56, 0.9) | 0.64 |
| No\_care : Nfemales : Nmales\_3 vs Care : Nfemales : Nmales\_3 | 0.27 (-0.23, 0.91) | 0.22 |
| No\_care : Nfemales : Nmales\_1 vs Care : Nfemales : Nmales\_1 | 0.24 (-0.48, 1.2) | 0.448 |
| Care : Nfemales : Nmales\_3 vs Care : Nfemales : Nmales\_1 | 0.17 (-0.64, 0.8) | 0.69 |
| No\_care : Nfemales^2 : Nmales\_3 vs No\_care : Nfemales^2 : Nmales\_1 | 0.57 (-0.19, 1.33) | 0.102 |
| No\_care : Nfemales^2 : Nmales\_3 vs Care : Nfemales^2 : Nmales\_3 | -0.4 (-0.94, 0.26) | 0.258 |
| No\_care : Nfemales^2 : Nmales\_1 vs Care : Nfemales^2 : Nmales\_1 | -0.42 (-1.45, 0.32) | 0.198 |
| Care : Nfemales^2 : Nmales\_3 vs Care : Nfemales^2 : Nmales\_1 | 0.43 (-0.41, 1.19) | 0.342 |
| Random Effects | Posterior Mode (CI) | I2 % (CI) |
| Year | 0.01 (0, 0.19) | 1.29 (0.01, 5.23) |
| Camp | 0.41 (0, 1.09) | 14.62 (0.52, 27.84) |
| Residual | 3.07 (2.32, 3.66) | 84.09 (69.83, 98.91) |

# **P**: The effect of average within-group relatedness on the number of chicks produced by males

| Fixed Effects | Posterior Mode (CI) | pMCMC |
| --- | --- | --- |
| Male relatedness | -0.27 (-0.65, 0.21) | 0.3 |
| No\_care | -0.42 (-2.08, 0.37) |  |
| Care | -1.66 (-3, -0.32) |  |
| No\_care : Nfemales | 0.56 (0.25, 1.06) |  |
| Care : Nfemales | 0.54 (0.17, 0.97) |  |
| No\_care : Nfemales^2 | -0.25 (-0.61, 0.08) |  |
| Care : Nfemales^2 | 0.11 (-0.26, 0.52) |  |
| No\_care : Male relatedness | 0.01 (-0.57, 0.44) | 0.788 |
| Care : Male relatedness | -0.19 (-1.16, 0.32) | 0.212 |
| Fixed Effect Comparisons | Posterior Mode (CI) | pMCMC |
| No\_care : Male relatedness vs Care : Male relatedness | 0.33 (-0.48, 1.31) | 0.352 |
| Random Effects | Posterior Mode (CI) | I2 % (CI) |
| Year | 0 (0, 0.09) | 18.09 (0.07, 68.46) |
| Camp | 0 (0, 0.46) | 63.74 (5.62, 99.68) |
| Residual | 0 (0, 0.07) | 18.17 (0.09, 70.59) |

# **Q**: The effect of average within-group relatedness on the number of chicks produced by females

| Fixed Effects | Posterior Mode (CI) | pMCMC |
| --- | --- | --- |
| Female relatedness | -0.24 (-0.5, 0.08) | 0.122 |
| No\_care : Nmales\_3 | 0.45 (-0.39, 1.32) |  |
| No\_care : Nmales\_1 | 0.66 (-0.27, 1.51) |  |
| Care : Nmales\_3 | -0.45 (-1.25, 0.43) |  |
| Care : Nmales\_1 | -0.83 (-1.62, 0.24) |  |
| No\_care : Nfemales | 0.48 (-0.37, 1.07) |  |
| Care : Nfemales | 0.33 (-0.74, 1.39) |  |
| No\_care : Nfemales^2 | -0.36 (-0.95, 0.17) |  |
| Care : Nfemales^2 | 0.07 (-0.77, 0.92) |  |
| No\_care : Female relatedness | -0.36 (-0.62, 0.09) | 0.11 |
| Care : Female relatedness | 0.04 (-0.52, 0.56) | 0.952 |
| Fixed Effect Comparisons | Posterior Mode (CI) | pMCMC |
| No\_care : Female relatedness vs Care : Female relatedness | -0.2 (-0.91, 0.32) | 0.33 |
| Random Effects | Posterior Mode (CI) | I2 % (CI) |
| Year | 0 (0, 0.06) | 6.48 (0.05, 26.22) |
| Camp | 0.1 (0.03, 0.36) | 70.85 (31.52, 98.98) |
| Residual | 0 (0, 0.18) | 22.67 (0.23, 62.04) |

# **R**: Sample size of experiment and summary statistics of reproductive success and incubation

|  | 2012 (N=11) | 2013 (N=11) | 2014 (N=12) | 2015 (N=14) | 2016 (N=17) | 2017 (N=15) | 2018 (N=16) | Total (N=96) |
| --- | --- | --- | --- | --- | --- | --- | --- | --- |
| **Number of females** |  |  |  |  |  |  |  |  |
| 1 | 2 (18.2%) | 2 (18.2%) | 2 (16.7%) | 4 (28.6%) | 4 (23.5%) | 4 (26.7%) | 4 (25.0%) | 22 (22.9%) |
| 3 | 4 (36.4%) | 4 (36.4%) | 4 (33.3%) | 4 (28.6%) | 5 (29.4%) | 4 (26.7%) | 4 (25.0%) | 29 (30.2%) |
| 4 | 5 (45.5%) | 5 (45.5%) | 6 (50.0%) | 0 (0.0%) | 0 (0.0%) | 0 (0.0%) | 0 (0.0%) | 16 (16.7%) |
| 6 | 0 (0.0%) | 0 (0.0%) | 0 (0.0%) | 6 (42.9%) | 8 (47.1%) | 7 (46.7%) | 8 (50.0%) | 29 (30.2%) |
| **Number of males** |  |  |  |  |  |  |  |  |
| 1 | 6 (54.5%) | 6 (54.5%) | 6 (50.0%) | 2 (14.3%) | 3 (17.6%) | 3 (20.0%) | 4 (25.0%) | 30 (31.2%) |
| 3 | 5 (45.5%) | 5 (45.5%) | 6 (50.0%) | 12 (85.7%) | 14 (82.4%) | 12 (80.0%) | 12 (75.0%) | 66 (68.8%) |
| **Group composition (No.Males No.females)** |  |  |  |  |  |  |  |  |
| 1 1 | 2 (18.2%) | 2 (18.2%) | 2 (16.7%) | 0 (0.0%) | 0 (0.0%) | 0 (0.0%) | 0 (0.0%) | 6 (6.2%) |
| 1 3 | 2 (18.2%) | 2 (18.2%) | 2 (16.7%) | 0 (0.0%) | 0 (0.0%) | 0 (0.0%) | 0 (0.0%) | 6 (6.2%) |
| 1 4 | 2 (18.2%) | 2 (18.2%) | 2 (16.7%) | 0 (0.0%) | 0 (0.0%) | 0 (0.0%) | 0 (0.0%) | 6 (6.2%) |
| 1 6 | 0 (0.0%) | 0 (0.0%) | 0 (0.0%) | 2 (14.3%) | 3 (17.6%) | 3 (20.0%) | 4 (25.0%) | 12 (12.5%) |
| 3 1 | 0 (0.0%) | 0 (0.0%) | 0 (0.0%) | 4 (28.6%) | 4 (23.5%) | 4 (26.7%) | 4 (25.0%) | 16 (16.7%) |
| 3 3 | 2 (18.2%) | 2 (18.2%) | 2 (16.7%) | 4 (28.6%) | 5 (29.4%) | 4 (26.7%) | 4 (25.0%) | 23 (24.0%) |
| 3 4 | 3 (27.3%) | 3 (27.3%) | 4 (33.3%) | 0 (0.0%) | 0 (0.0%) | 0 (0.0%) | 0 (0.0%) | 10 (10.4%) |
| 3 6 | 0 (0.0%) | 0 (0.0%) | 0 (0.0%) | 4 (28.6%) | 5 (29.4%) | 4 (26.7%) | 4 (25.0%) | 17 (17.7%) |
| **Number of eggs laid during period without care** |  |  |  |  |  |  |  |  |
| Mean (SD) | 79.27 (36.32) | 110.09 (67.75) | 122.42 (46.99) | 114.57 (68.91) | 130.76 (69.94) | 140.60 (95.47) | 150.69 (80.44) | 123.95 (71.80) |
| Range | 21.00 - 121.00 | 9.00 - 188.00 | 49.00 - 217.00 | 16.00 - 222.00 | 31.00 - 266.00 | 16.00 - 282.00 | 29.00 - 302.00 | 9.00 - 302.00 |
| **Number of eggs laid during period with care** |  |  |  |  |  |  |  |  |
| Mean (SD) | 22.45 (12.67) | 27.36 (11.21) | 20.50 (9.25) | 24.29 (14.78) | 21.18 (11.76) | 24.87 (15.92) | 15.56 (7.64) | 22.04 (12.35) |
| Range | 0.00 - 42.00 | 8.00 - 43.00 | 9.00 - 37.00 | 8.00 - 63.00 | 0.00 - 43.00 | 8.00 - 61.00 | 5.00 - 29.00 | 0.00 - 63.00 |
| **Number of chicks hatched during period without care** |  |  |  |  |  |  |  |  |
| Mean (SD) | 35.27 (24.42) | 46.82 (40.90) | 69.08 (42.67) | 42.00 (31.23) | 53.65 (34.81) | 68.53 (64.25) | 55.69 (50.95) | 53.66 (44.00) |
| Range | 1.00 - 74.00 | 0.00 - 102.00 | 9.00 - 161.00 | 7.00 - 106.00 | 0.00 - 144.00 | 3.00 - 197.00 | 1.00 - 214.00 | 0.00 - 214.00 |
| **Number of chicks hatched during period with care** |  |  |  |  |  |  |  |  |
| Mean (SD) | 3.00 (3.35) | 2.09 (2.81) | 2.58 (4.19) | 5.93 (6.38) | 3.41 (2.67) | 3.53 (4.47) | 2.00 (2.99) | 3.26 (4.09) |
| Range | 0.00 - 9.00 | 0.00 - 7.00 | 0.00 - 10.00 | 0.00 - 23.00 | 0.00 - 10.00 | 0.00 - 12.00 | 0.00 - 12.00 | 0.00 - 23.00 |
| **Number of broken eggs** |  |  |  |  |  |  |  |  |
| Mean (SD) | 12.36 (10.40) | 15.09 (8.28) | 13.17 (9.57) | 8.29 (4.71) | 10.12 (9.07) | 12.47 (7.31) | 6.25 (5.87) | 10.78 (8.21) |
| Range | 0.00 - 30.00 | 1.00 - 30.00 | 4.00 - 35.00 | 4.00 - 19.00 | 0.00 - 32.00 | 1.00 - 24.00 | 1.00 - 21.00 | 0.00 - 35.00 |
| **Proportion of time incubating** |  |  |  |  |  |  |  |  |
| Mean (SD) | 0.30 (0.30) | 0.36 (0.30) | 0.48 (0.24) | 0.47 (0.26) | 0.49 (0.26) | 0.45 (0.32) | 0.63 (0.23) | 0.47 (0.28) |
| Range | 0.00 - 0.83 | 0.00 - 0.83 | 0.09 - 0.82 | 0.06 - 0.72 | 0.00 - 0.93 | 0.01 - 0.88 | 0.16 - 0.96 | 0.00 - 0.96 |

# **S**: Overview of replacements and removals of individuals in experimental groups

|  | | | | Before replacement/removal | | After replacement/removal | |
| --- | --- | --- | --- | --- | --- | --- | --- |
| Year | Group ID | Date | Sex of removed | no. males | no. females | no. males | no. females |
| **Replacements** | | | | | | | |
| 2013 | 108 | 2013-09-26 | Male | 1 | 3 | 1 | 3 |
| 2014 | 58 | 2014-05-20 | Female | 1 | 4 | 1 | 4 |
| 2014 | 63 | 2014-08-09 | Male | 3 | 3 | 3 | 3 |
| 2015 | 58 | 2015-06-23 | Male | 3 | 1 | 3 | 1 |
| 2016 | 20 | 2016-07-28 | Female | 1 | 6 | 1 | 6 |
| 2016 | 60 | 2016-09-29 | Female | 3 | 3 | 3 | 3 |
| 2016 | 70 | 2016-05-17 | Male | 3 | 3 | 3 | 3 |
| **Removals** | | | | | | | |
| 2014 | 58 | 2014-08-28 | Female | 1 | 4 | 1 | 3 |
| 2015 | 25 | 2015-12-01 | Male | 3 | 6 | 2 | 6 |
| 2015 | 27 | 2015-10-29 | Male | 3 | 6 | 2 | 6 |
| 2015 | 60 | 2015-10-30 | Male | 3 | 3 | 2 | 3 |
| 2015 | 64 | 2015-07-21 | Female | 1 | 6 | 1 | 5 |
| 2016 | 25 | 2016-08-07 | Female | 3 | 6 | 3 | 5 |
| 2016 | 27 | 2016-11-14 | Female | 3 | 6 | 3 | 5 |
| 2016 | 70 | 2016-12-02 | Male | 3 | 3 | 2 | 3 |
| 2017 | 22 | 2017-10-27 | Female | 3 | 3 | 3 | 2 |
| 2017 | 29 | 2017-11-13 | Female | 3 | 1 | 3 | 0 |
| 2017 | 62 | 2017-09-16 | Male | 3 | 6 | 2 | 6 |
| 2018 | 25 | 2018-11-18 | Female | 3 | 6 | 3 | 5 |

# R Session Information

```
## R version 4.1.1 (2021-08-10)
## Platform: x86_64-apple-darwin17.0 (64-bit)
## Running under: macOS Big Sur 10.16
## 
## Matrix products: default
## BLAS:   /Library/Frameworks/R.framework/Versions/4.1/Resources/lib/libRblas.0.dylib
## LAPACK: /Library/Frameworks/R.framework/Versions/4.1/Resources/lib/libRlapack.dylib
## 
## locale:
## [1] en_GB.UTF-8/en_GB.UTF-8/en_GB.UTF-8/C/en_GB.UTF-8/en_GB.UTF-8
## 
## attached base packages:
## [1] stats     graphics  grDevices utils     datasets  methods   base     
## 
## other attached packages:
##  [1] ggpubr_0.4.0               magick_2.7.3              
##  [3] devtools_2.4.3             usethis_2.1.5             
##  [5] arsenal_3.6.3              ggsci_2.9                 
##  [7] data.table_1.14.2          kableExtra_1.3.4          
##  [9] stringdist_0.9.8           openxlsx_4.2.5            
## [11] wesanderson_0.3.6          arm_1.12-2                
## [13] lme4_1.1-28                PerformanceAnalytics_2.0.4
## [15] xts_0.12.1                 zoo_1.8-9                 
## [17] sciplot_1.2-0              gplots_3.1.1              
## [19] car_3.0-12                 carData_3.0-5             
## [21] reshape_0.8.8              Hmisc_4.7-0               
## [23] Formula_1.2-4              survival_3.3-1            
## [25] QuantPsyc_1.5              MASS_7.3-56               
## [27] boot_1.3-28                languageR_1.5.0           
## [29] doBy_4.6.12                MCMCglmm_2.33             
## [31] coda_0.19-4                Matrix_1.4-1              
## [33] cowplot_1.1.1              ape_5.6-2                 
## [35] forcats_0.5.1              stringr_1.4.0             
## [37] purrr_0.3.4                readr_2.1.2               
## [39] tidyr_1.2.0                tibble_3.1.6              
## [41] ggplot2_3.3.5              tidyverse_1.3.1           
## [43] dplyr_1.0.8                Rmisc_1.5                 
## [45] plyr_1.8.7                 lattice_0.20-45           
## [47] knitr_1.38                
## 
## loaded via a namespace (and not attached):
##   [1] readxl_1.4.0         backports_1.4.1      systemfonts_1.0.4   
##   [4] splines_4.1.1        digest_0.6.29        htmltools_0.5.2     
##   [7] fansi_1.0.3          magrittr_2.0.3       checkmate_2.0.0     
##  [10] memoise_2.0.1        cluster_2.1.3        remotes_2.4.2       
##  [13] tzdb_0.3.0           modelr_0.1.8         svglite_2.1.0       
##  [16] askpass_1.1          prettyunits_1.1.1    jpeg_0.1-9          
##  [19] colorspace_2.0-3     rvest_1.0.2          haven_2.4.3         
##  [22] xfun_0.30            callr_3.7.0          crayon_1.5.1        
##  [25] microbenchmark_1.4.9 jsonlite_1.8.0       glue_1.6.2          
##  [28] gtable_0.3.0         webshot_0.5.2        pkgbuild_1.3.1      
##  [31] abind_1.4-5          scales_1.1.1         qpdf_1.1            
##  [34] DBI_1.1.2            rstatix_0.7.0        Rcpp_1.0.8.3        
##  [37] viridisLite_0.4.0    htmlTable_2.4.0      foreign_0.8-82      
##  [40] pdftools_3.1.1       htmlwidgets_1.5.4    httr_1.4.2          
##  [43] RColorBrewer_1.1-2   ellipsis_0.3.2       farver_2.1.0        
##  [46] pkgconfig_2.0.3      nnet_7.3-17          sass_0.4.1          
##  [49] dbplyr_2.1.1         utf8_1.2.2           labeling_0.4.2      
##  [52] tidyselect_1.1.2     rlang_1.0.2          munsell_0.5.0       
##  [55] cellranger_1.1.0     tools_4.1.1          cachem_1.0.6        
##  [58] cli_3.2.0            generics_0.1.2       pacman_0.5.1        
##  [61] broom_0.7.12         evaluate_0.15        fastmap_1.1.0       
##  [64] yaml_2.3.5           processx_3.5.3       fs_1.5.2            
##  [67] zip_2.2.0            caTools_1.18.2       nlme_3.1-157        
##  [70] xml2_1.3.3           brio_1.1.3           compiler_4.1.1      
##  [73] rstudioapi_0.13      curl_4.3.2           png_0.1-7           
##  [76] ggsignif_0.6.3       testthat_3.1.3       reprex_2.0.1        
##  [79] bslib_0.3.1          stringi_1.7.6        highr_0.9           
##  [82] ps_1.6.0             desc_1.4.1           cubature_2.0.4.4    
##  [85] nloptr_2.0.0         tensorA_0.36.2       vctrs_0.4.0         
##  [88] pillar_1.7.0         lifecycle_1.0.1      jquerylib_0.1.4     
##  [91] bitops_1.0-7         corpcor_1.6.10       R6_2.5.1            
##  [94] latticeExtra_0.6-29  KernSmooth_2.23-20   gridExtra_2.3       
##  [97] sessioninfo_1.2.2    pkgload_1.2.4        gtools_3.9.2        
## [100] assertthat_0.2.1     rprojroot_2.0.2      withr_2.5.0         
## [103] Deriv_4.1.3          mgcv_1.8-40          parallel_4.1.1      
## [106] hms_1.1.1            quadprog_1.5-8       grid_4.1.1          
## [109] rpart_4.1.16         minqa_1.2.4          rmarkdown_2.13      
## [112] lubridate_1.8.0      base64enc_0.1-3
```
